# Methylome Analysis in Chickens Immunized with Infectious Laryngotracheitis Vaccine

**DOI:** 10.1371/journal.pone.0100476

**Published:** 2015-06-24

**Authors:** José A. Carrillo, Yanghua He, Juan Luo, Kimberly R. Menendez, Nathaniel L. Tablante, Keji Zhao, Joseph N. Paulson, Bichun Li, Jiuzhou Song

**Affiliations:** 1 Department of Animal and Avian Sciences, University of Maryland, College Park, Maryland, United States of America; 2 Virginia-Maryland Regional College of Veterinary Medicine, University of Maryland, College Park, Maryland, United States of America; 3 Laboratory of Molecular Immunology, National Heart, Lung and Blood Institute, National Institutes of Health, Bethesda, Maryland, United States of America; 4 Center for Bioinformatics and Computational Biology, University of Maryland, College Park, Maryland, United States of America; 5 College of Animal Science and Technology, Yangzhou University, Yangzhou City, Jiangsu Province, P. R. China; Peking University Cancer Hospital and Institute, CHINA

## Abstract

In this study we investigated the methylome of chickens immunized with Infectious laryngotracheitis (ILT) vaccine derived from chicken embryos. Methyl-CpG binding domain protein-enriched genome sequencing (MBD-Seq) method was employed in the detection of the 1,155 differentially methylated regions (DMRs) across the entire genome. After validation, we ascertained the genomic DMRs distribution and annotated them regarding genes, transcription start sites (TSS) and CpG islands. We found that global DNA methylation decreased in vaccinated birds, presenting 704 hypomethylated and 451 hypermethylated DMRs, respectively. Additionally, we performed an enrichment analysis detecting gene networks, in which cancer and RNA post-transcriptional modification appeared in the first place, followed by humoral immune response, immunological disease and inflammatory disease. The top four identified canonical pathways were EIF2 signaling, regulation of EIF4 and p70S6K signaling, axonal guidance signaling and mTOR signaling, providing new insight regarding the mechanisms of ILT etiology. Lastly, the association between DNA methylation and differentially expressed genes was examined, and detected negative correlation in seventeen of the eighteen genes.

## Introduction

Infectious Laryngotracheitis (ILT) is a disease caused by the *Gallid herpesvirus 1 (GaHV-1)*. The disease spreads worldwide and presents a challenge for the poultry industry due to its detrimental economic impact. Morbidity varies from 50 to 100%, and mortality between 10 and 20%, reaching sometimes 70% of the flocks [1]. Mostly, ILT is associated with chicken in areas of high poultry production but the disease also affects other birds such pheasants, peafowls and turkeys [2]. After the incubation period of 6 to 12 days, infected birds could display different symptoms, such as conjunctivitis, sinusitis and nasal discharge, bloody mucous exudate, continuous head shaking and hyperextension of the neck [3]. The course of the disease extends from 10 to 14 days. Grave acute cases will die within a few days [3]. Although chickens could fully recover in the mild cases, the loss in growth, meat quality and egg yield is significant [4]. Therefore, the ILT is being listed as one of the priority poultry diseases in USA.

ILTV diagnostic is based on symptomatology, lesions, histology and laboratory analysis (PCR, ELISA) [5]. Vaccination is used to prevent the incidence of the disease in endemic regions or in emergency situations. Attenuated live vaccines are administered intraocular at the age of 4 to 6 weeks, and revaccination is normally performed at 14–16 weeks by drinking water [6]. Since inoculation could cause “vaccinal laryngotracheitis” in immune compromised individuals, only healthy birds should receive the antigen [7]. Modified-live vaccines have been linked to several problems including adverse vaccine reactions, increase virulence by successive passages of the virus from bird to bird, and the possible harbor of latent virus in vaccinated individuals [8]. All these reasons justify the avoidance of modified-live vaccines. Recently, a recombinant vaccine has been released for commercial use [9]. Recombinant vaccines have a promissory future because a single dose confers lifelong immunity and lack most of the undesirable reactions. Despite all the benefits described before, massive utilization of recombinant vaccine is still scarce. To devise improved methods for the diagnosis and control of the disease, more studies are necessary for understanding better the onset, establishment and course of the infection.

The ILTV presents a linear and double-stranded DNA. Its genome size is approximately 150 kb, containing 48% of GCs [3]. The genome is organized in unique short and long regions surrounded by identical internal and terminal repeat sequences, encoding 80 open reading frames [10]. So far, most of the studies in ILT have been focused on the viral molecular structure and differences among strains [11, 12]. Identification of those divergences permits to recognize new viral mutations with atypical clinical signs that redefine the known course of the disease [4].

Obviously, the establishment of the infection involves an interaction between the virus and the host. Nevertheless, many aspects from the host side are still unknown or superficially described, although studies *in vitro* explored genomic host responses to virulent and vaccine ILTVs in lung cells obtained from chicken embryos [13, 14]. Comparing vaccine and virulent ITL, they only identified several differentially expressed genes, such as *C8orf79*, *F10*, *NPY* and *BMP2* [13]. However, similar research *in vivo* exploration has not yet been reported. Although identification of genes explains part of the implicated immunological mechanisms, the epigenetic regulation of ILTV infection is still unrevealed. As we know, it is essential to understand in details the host response to vaccination and infection for potential development of novel vaccines, which will be important to control ILT.

DNA methylation—a common epigenetic phenomenon—consists on the addition of a methyl group to the cytosine or adenine DNA nucleotides. In mammals, DNA methylation mostly occurs in CpG sites and ranges from 60 to 90 percent [15, 16]. It has been associated with repressive gene stage and plays a major role in many biological processes such as cells differentiation, carcinogenesis, genomic imprinting, chromosome inactivation and expression regulation [17]. Unmethylated CpGs aggregate in clusters denominated CpG islands, which exist in the 5’ regulatory regions of many genes [18]. Notably, diagnostic and prevention of tumors for different tissues were developed based on their methylation profile, indicating that methylation pattern differs between normal and cancerous tissues [19, 20].

In this study we explored the DNA methylation profiles and identified differentially methylated regions (DMRs) between ILTV vaccinated and control chicken groups, using the methyl-CpG binding domain protein-enriched genome sequencing (MBD-Seq) method [21]. Subsequently, we annotated the DMRs regarding their location to genes and CpG islands, and performed enrichment and pathways analyses. Finally, we found some genes that were differentially expressed in RNA-Seq analysis and also overlapped with the identified DMRs associated to its correspondent promoter, and explored the relationship between DNA methylation and gene expression.

## Results

Twelve libraries were constructed from the birds’ DNAs. Each condition (control and vaccinated) has three elute concentrations with two replications. Before euthanasia, the birds were checked and all individuals from the vaccinated group showed mild to acute clinical signs. The respective scores ranged from 0 to 3 and none of the birds received more than 3 during the experiment. Accordingly, the control group scored 0 for all individuals. Two biological replicates from each condition were used to extract tracheal DNA for the MBD-Seq analysis. Table 1 shows the number of reads per sample, in which the control with high elute concentration has the less number of aligned reads, oppositely to the vaccinated and low concentration that holds the largest number of aligned fragments. The percentage of alignment for all samples ranged from 66.11 to 83.69 percent. Considering the samples by conditions (Control-Vaccinated) and replicates (1–2), and comparing individual samples within groups, it is consistent that high elute concentration produces DNA fragments that align in a smaller proportion to the reference genome.

**Table 1:** Sequencing and mapping details of the samples.

Sample	Reads Processed	Aligned Reads	Unaligned Reads	Aligned (%)
LowControl1	15,840,740	12,242,921	3,597,819	77.29
MedControl1	12,049,016	9,408,739	2,640,277	78.09
HighControl1	17,685,076	11,692,413	5,992,663	66.11
LowControl2	7,788,797	6,213,214	1,575,583	79.77
MedControl2	7,406,875	5,497,846	1,909,029	74.23
HighControl2	18,880,981	12,546,980	6,334,001	66.45
LowVac1	7,475,829	6,256,266	1,219,563	83.69
MedVac1	7,389,194	5,849,164	1,540,030	79.16
HighVac1	9,922,376	6,909,168	3,013,208	69.63
LowVac2	8,402,205	6,992,132	1,410,073	83.22
MedVac2	6,437,102	5,335,687	1,101,415	82.89
HighVac2	7,383,856	5,066,831	2,317,025	68.62

For peaks detection, Table 2 shows the samples and the last column arrays the numbers of peaks for each sample. This is helpful to compare the numbers of peaks between replicates, demonstrating how well replications represent their corresponding biological condition. Samples with high salt concentration have the lowest number of peaks, ranging from 7,858 to 11,109. Medium and High concentrations behaved similarly with comparable peak numbers for three members of these groups. Low concentration samples clustered in two groups of similar values.

**Table 2:** Description and number of peaks per sample.

ID	Tissue	Condition	Treatment	Replicate	Peak caller	Intervals
HC1	Trachea	Control	High	1	Macs	8,362
HC2	Trachea	Control	High	2	Macs	7,858
HV1	Trachea	Vaccinated	High	1	Macs	11,109
HV2	Trachea	Vaccinated	High	2	Macs	9,571
MC1	Trachea	Control	Medium	1	Macs	30,202
MC2	Trachea	Control	Medium	2	Macs	21,891
MV1	Trachea	Vaccinated	Medium	1	Macs	30,036
MV2	Trachea	Vaccinated	Medium	2	Macs	29,909
LC1	Trachea	Control	Low	1	Macs	31,995
LC2	Trachea	Control	Low	2	Macs	24,667
LV1	Trachea	Vaccinated	Low	1	Macs	24,833
LV2	Trachea	Vaccinated	Low	2	Macs	28,398

**Fig 1:**
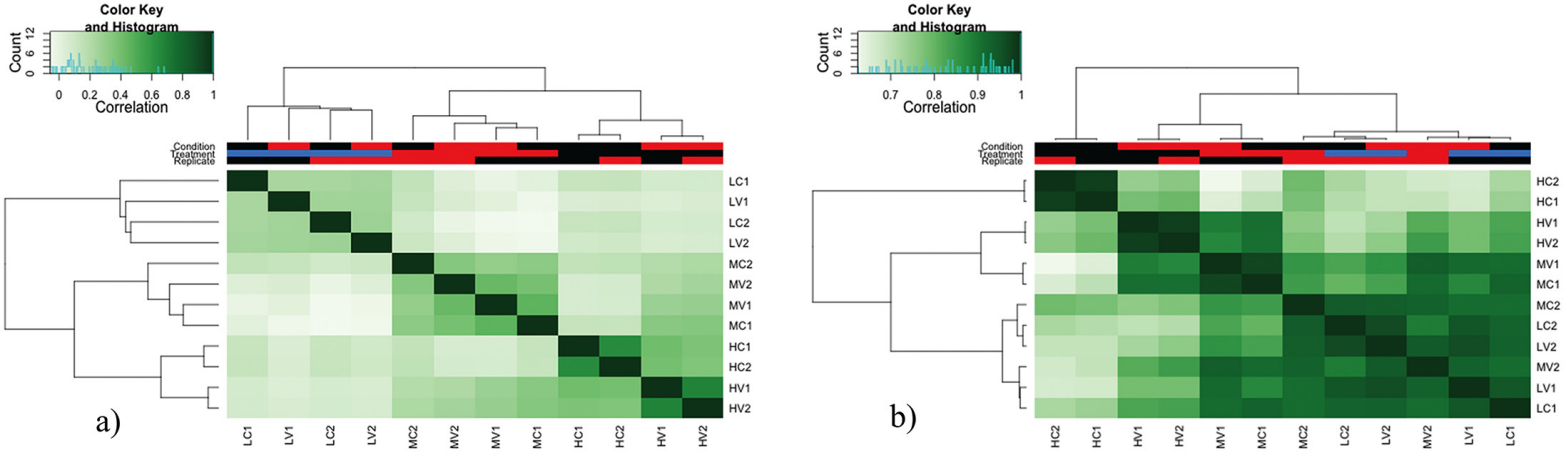
Heat-maps obtained at different stages of the analysis a) Occupancy analysis result based on rows cross-correlations from the peaks detected by MACS. The dendogram describes the similarity of peaks among samples and serves as a quality control for the peak calling step b) Clustering employing the reads counts of each sample in the entire set of potential binding sites, which are represented by rows in the matrix.

To evaluate the data quality, we did occupancy analysis based on the cross-correlations of each row using only the peak calling data (Fig 1a), indicating the similarity of peak scores and serving as a technical quality control as well. The samples clustered based on concentration instead on replication, excepting for the high concentration samples where they paired by replication and condition (HC1-HC2 and HV1-HV2), as is reflected in the larger correlation coefficient among these samples. Then, a binding matrix was generated using the read counts instead of the previously used peak confidence scores (Fig 1b). The results show affinity scores correlation (reads counts) in all possible binding locations. For most of the samples correlations are higher than in Fig 1a. Obviously, this approach worked well for grouping high concentration samples by condition and replicates but failed to classify all samples based on condition.

**Fig 2:**
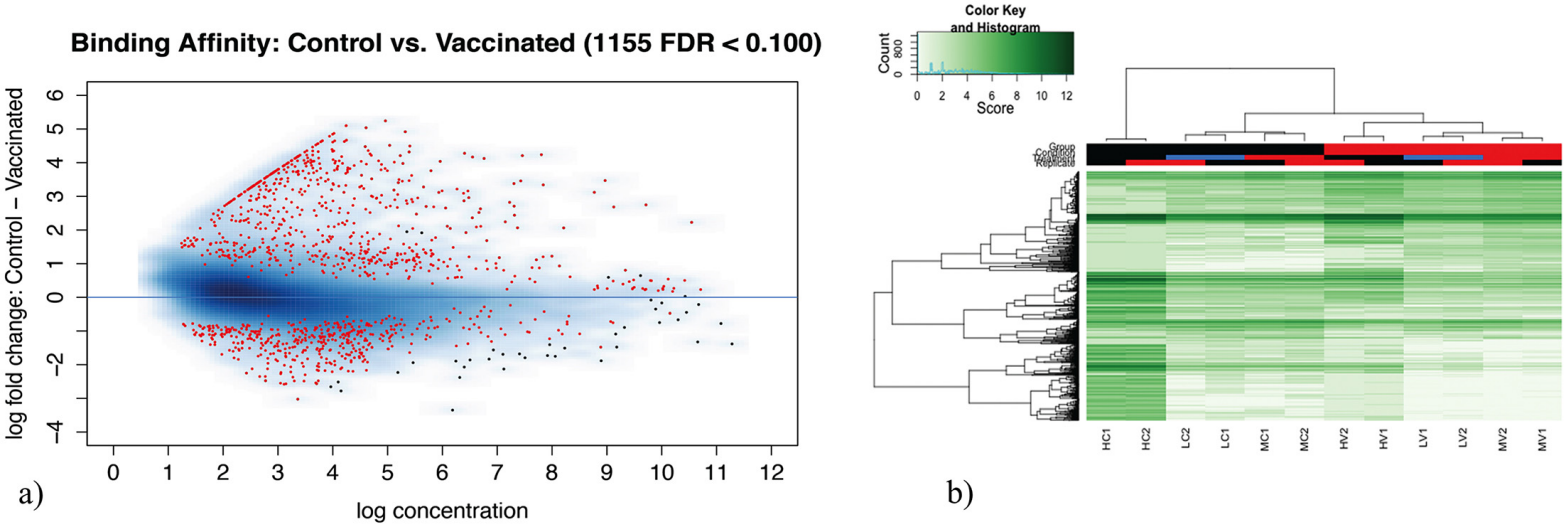
Differentially methylated regions visualization and cluster of samples considering their methylation levels a) The MA plot shows in red the differentially methylated regions obtained with a false discovery rate of < 0.1 b) Heat-map of samples demonstrates a perfect classification of the condition of the individuals based on methylation levels in the differentially methylated regions.

By using DiffBind, we defined a contrast based on conditions and detected 85,493 unique peaks and 47,356 overlapped peaks. The resulting matrix of 47,356 x 12 represents binding locations and number of samples for rows and columns, respectively. We detected 1,155 DMRs across the entire chicken genome for birds that were inoculated with ILT chicken embryo origin vaccine. The differentially methylated regions (DMRs) (FDR<0.1) are presented as red dots in the Fig 2a. We found that 451 and 704 DMRs were hypomethylated and hypermethylated in the vaccinated group, respectively. The clustering of samples, using only the identified DMRs, was shown in the Fig 2b. The extensive list of DMRs is provided as an excel spreadsheet in the S2 Table. The results coincide with the condition of the samples, especially for the vaccinated samples that were assigned to the same cluster, suggesting that these DNA varying methylation levels could be used to predict the outcome of the individual.

**Fig 3:**
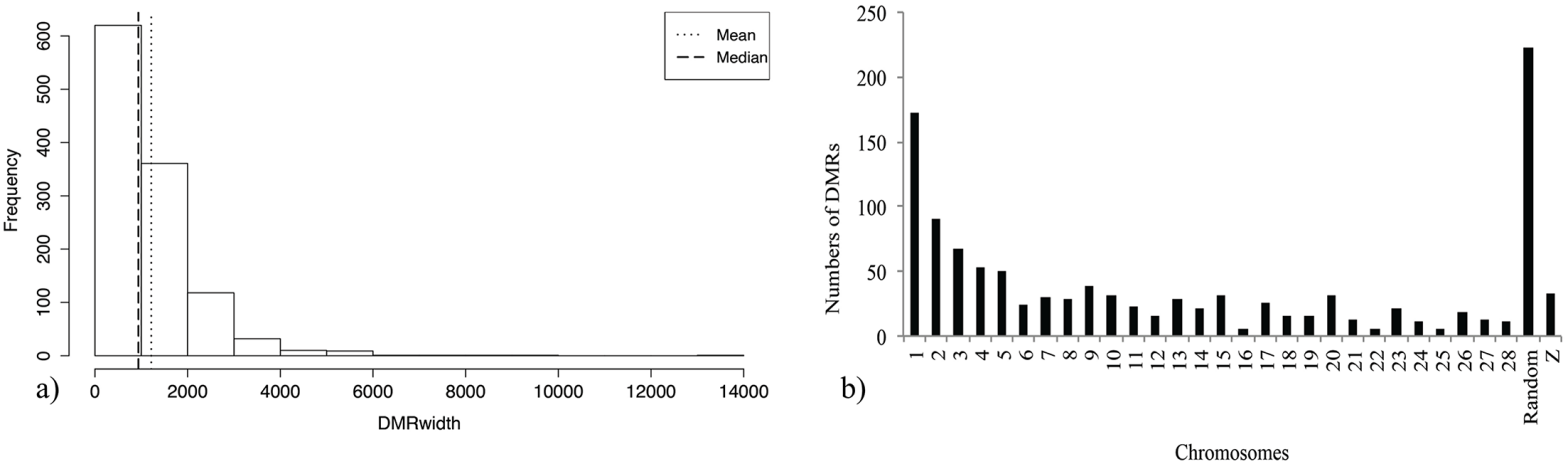
Length and chromosomal location frequencies of the DMRs a) DMRs' length density. The abscissa represents the extent of the DMRs in base pairs. The dashed and dotted lines correspond for the median and mean respectively b) Distribution of the Differentially Methylated Regions per chromosome without normalization (ignoring chromosome length).

As for the distribution of the DMRs’ length, as shown in Fig 3a, we found the average was 1,215 with extreme values of 80 and 13,200 base pairs, respectively. Approximately more than 55% of the DMRs were less than 1,000 bp and only one percent of the DMRs accounted for fragments longer than 4,000 bp. Fig 3b illustrates the number of DMRs per chromosome. Due to the large number of unassigned locations in the chicken genome, the random chromosome accounted for 222 regions, followed by chromosome one with 172 DMRs. Chromosomes 16, 22 and 25 had the lowest numbers of DMRs with values of 5, 5 and 6, correspondingly.

**Fig 4:**
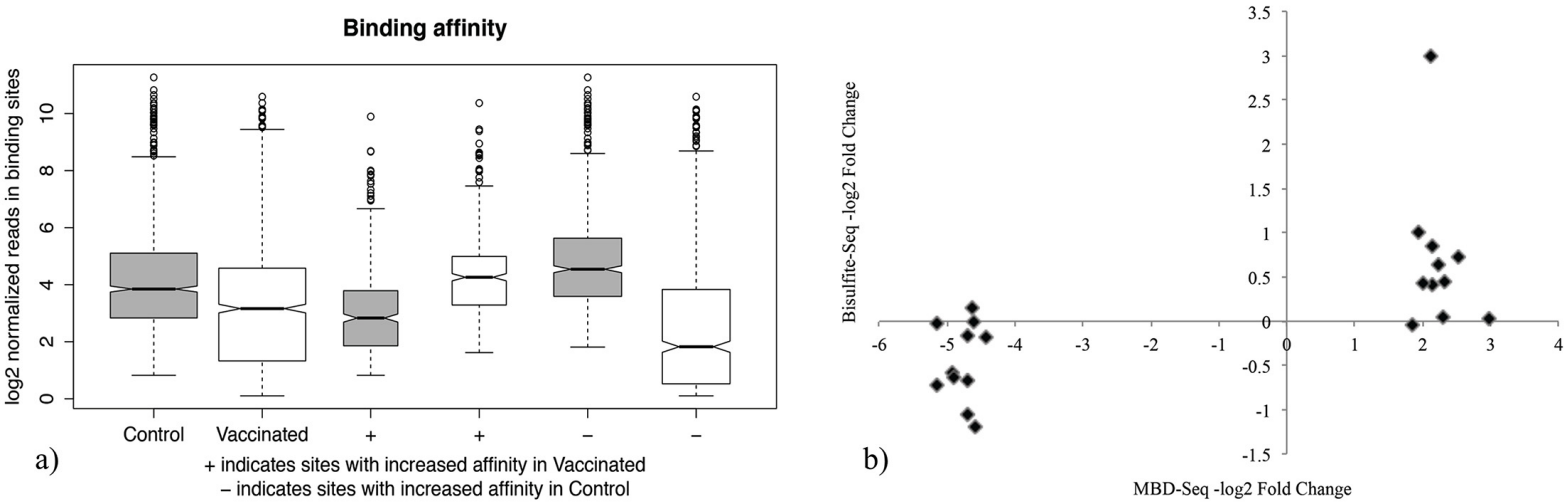
Binding sites reads counts and comparison of bisulfite sequencing and MBD-Seq approaches a) Normalized reads in binding sites per condition. The first two boxplots represent the overall methylation level in the control and vaccinated groups. The boxplots marked with the + and - signs correspond to the DMRs with enriched methylation levels in the vaccinated and control clusters, accordingly b) DNA methylation levels expressed in -log2 fold change from bisulfite sequencing (y-axis) and MBD-Seq (x-axis) methods.

The binding affinity between conditions was shown in the Fig 4a. We found that global DNA methylation decreased in vaccinated individuals. The + sign marked the 451 sites with increased affinity in the vaccinated group and the—sign for the 704 regions that also augmented its binding, but in the control cluster. The gray and white boxes corresponded to control and vaccinated within + or—groups, correspondingly. To test whether the distribution of the values was significantly different among them, a two-sided Wilcoxon-Mann-Whitney test was applied. From the Table 3, all were significant different (p<0.05), excepting (Control.DB+ versus Vacc.DB).

**Table 3:** Wilcoxon-Mann-Whiney test for the differentially methylated regions.

	Cont.DB	Vacc.DB	ContDB+	Vacc.DB+	Cont.DB-	Vacc.DB-
Cont.DB	1	1.06e-43	1.47e-29	3.13e-03	4.49e-17	1.46e-67
Vacc.DB	1.06e-43	1	2.83e-01	1.33e-25	2.15e-57	6.93e-15
Cont.DB+	1.47e-29	2.83e-01	1	1.28e-75	3.50e-65	7.40e-14
Vacc.DB+	3.13e-03	1.33e-25	1.28e-75	1	3.54e-06	3.17e-56
Cont.DB-	4.49e-17	2.15e-57	3.50e-65	3.54e-06	1	6.39e-117
Vacc.DB-	1.46e-67	6.93e-15	7.40e-14	3.17e-56	6.39e-117	1

**Fig 5:**
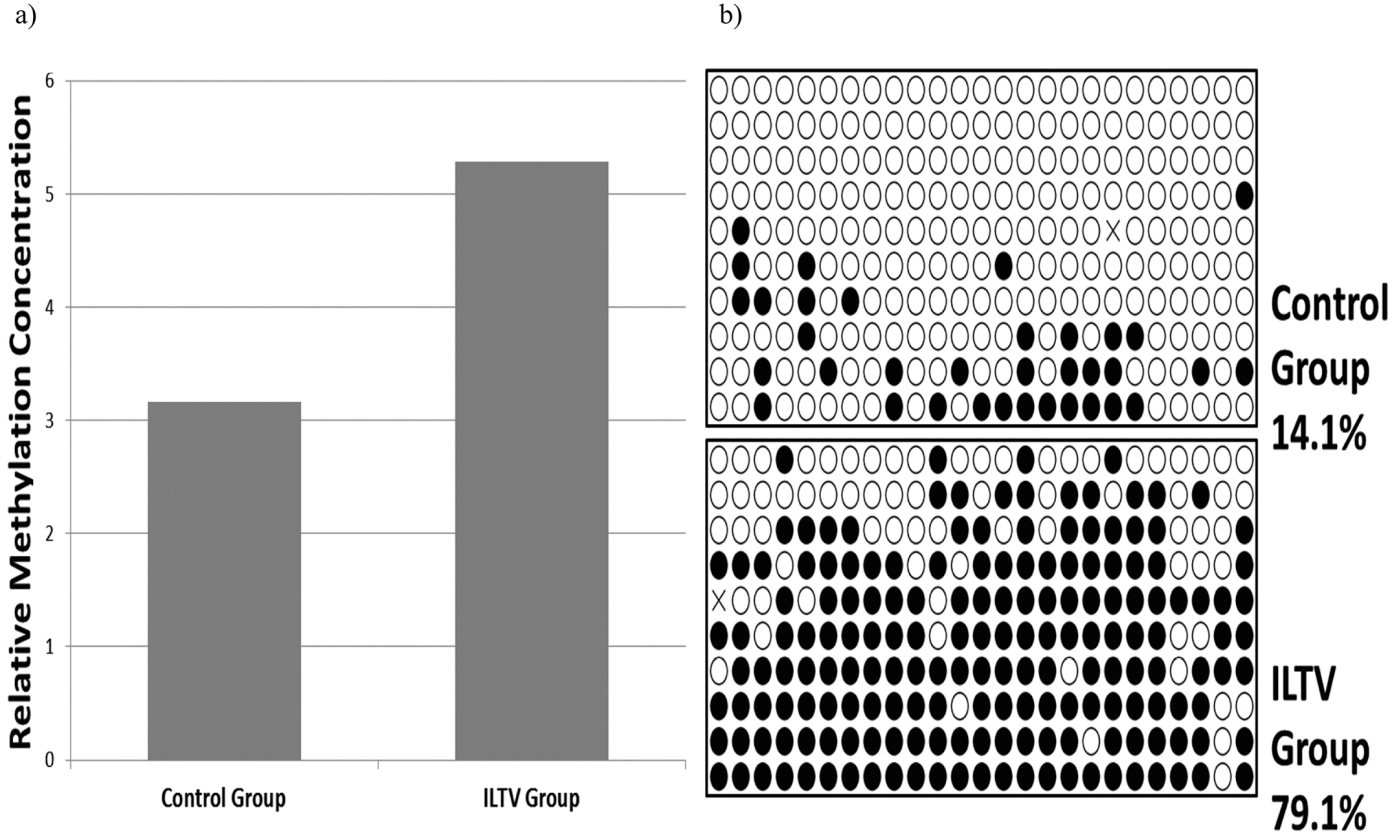
Bisulfite sequencing validation of MBD-Seq result (e.g., MBD22 region) a) Methylation concentration levels from MBD-seq b) Bisulfite sequencing results. Each line represents a plasmid sequence and each dot indicates a CpG site. An open circle indicates an unmethylated CpGs and a black dot methylated CpGs. The double-cross shape was the mutation. The methylation level was calculated as the number of methylated CpG sites divided by the total detected CpGs (mutation excluded).

In order to assess the reliability and accuracy of MBD-seq in DMRs detection, 36 DMRs were arbitrarily selected for validation. At least 10 colonies were cultured and then sequenced from each group. Twenty-two of those 36 DMRs (61%) had been sequenced. The calculated methylation levels are shown in the Fig 4b. We found that methylation levels of 19 of these 22 DMRs (86.4%) agreed with the directionality of the change revealed by MBD-seq, although only 36.8% of DMRs had significant differences (p< 0.01), detected by bisulfite sequencing. This technic employs only a segment of the DMR to perform the validation, thus the orientation of the change is more descriptive than the magnitude of methylation differences. For example, the methylation level of ILTV group was extremely higher than the control group for the MBD22 region (FDR = 0.016), employing the MBD-Seq method. In this case, the MBD-Seq result was accurate validated by the bisulfite sequencing approach (Fig 5).

**Fig 6:**
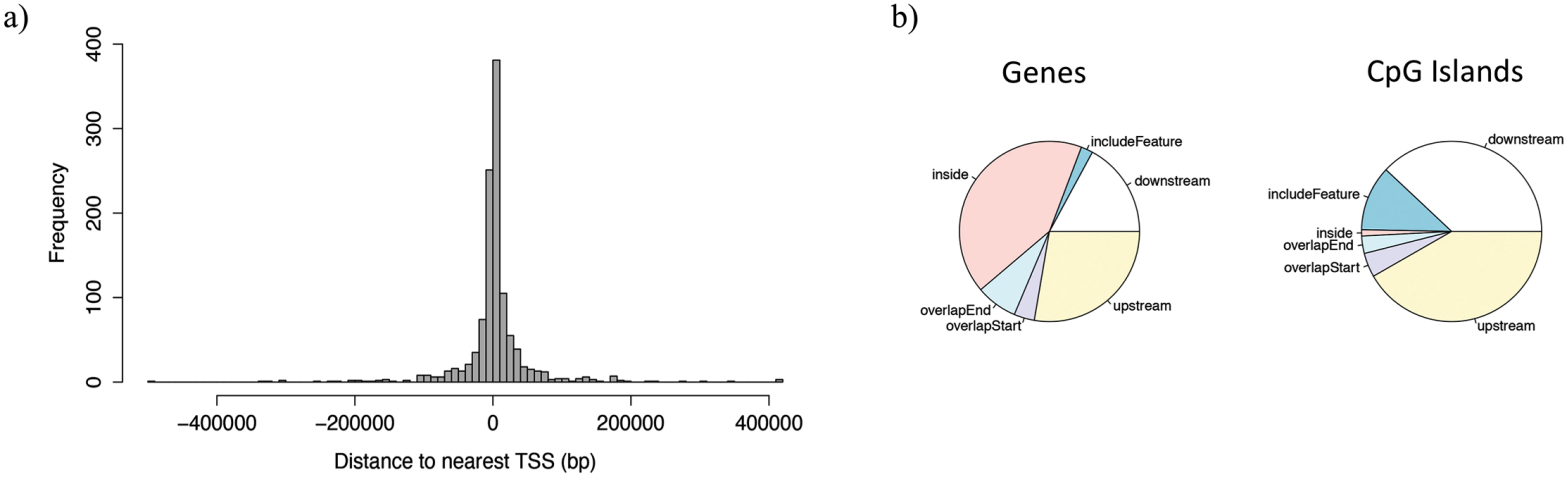
Frequency of DMRs’ distances to closest Transcription Start Site and DMRs location distribution regarding genes and CpG islands. a) The distance from the transcription start site is represented in base pairs from the 0 in the x-axis b) The labels in both pies represent: inside, DMR contained within the feature; include feature, the genomic feature is entirely included in the DMR; overlap start, the DMR extend over the start site of the feature; overlap end, the ending site of the feature overlaps with the DMR; downstream, DMR locates downstream the feature; upstream, the DMR aligns upstream the genomic feature.

The DMR annotation was subsequently performed (S3 Table). The distribution of DMRs’ distances to the closest Transcription Start Site (TSS) can be seen in the Fig 6a, demonstrating that approximately 60% of the DMRs are located within a range of 10,000 bp from the TSS. Moreover, DMRs location related to genes and CpG islands are summarized in the Fig 6b. We found that almost half of the DMRs are contained within genes and a quart in regions upstream the TSS; fragments located behind the end of the genes account for 16%. Because the chicken genome is well annotated and considers small genes, 30 of these reside within DMRs (includeFeature). Regarding CpG islands, as shown in the Fig 6b, the situation is different, 76% are outside the GpG island boundaries and 12% of the annotated CpG islands are included in the DMRs. Because CpG islands are normally unmethylated and have been found inside or near approximately 40% of mammalian promoter regions, i.e., most of DMRs are located upstream and downstream of CpG islands.

There is evidence that some promoters can initiate transcription in both directions, affecting the expression of more than one protein coding gene [22]. For this reason, we explored the differentially methylated regions to identified genes with bi-directional promoters, using 5,000 bp as the parameter for maximum distance. From the 1,155 identified DMRs, 165 are located within bi-directional promoters, accounting for 14.2% of the total. The list of peaks associated with bi-directional promoters is provided (S4 Table).

Using the genes identified from the annotated peaks, gene ontology enrichments and Ingenuity Pathway Analysis (IPA) were performed. The resultant top-notch networks were: a) Cancer, hematological disease, RNA pot-transcriptional modification; b) Hematological system development and function, humoral immune response, cellular assembly and organization; c) Hematological disease, immunological disease, inflammatory disease; d) Protein synthesis, cancer, hematological disease; e) Molecular transport, protein trafficking, cell cycle. Regarding molecular and cellular functions, the distinguished were: a) Cellular growth and proliferation; b) Cell death and survival; c) Cellular assembly and organization; d) Cellular function and maintenance; e) Cellular development. The top canonical pathways with its correspondent p-value and ratio are summarized in the Table 4. According to the p-value, the most significant ones are for EIF2 Signaling and regulation of eIF4 and p70S6K signaling pathways. PI3K Signaling in B Lymphocytes shows the highest p-value among the top five canonical functional pathways.

**Table 4:** Canonical Pathways identified using IPA analysis.

Pathway Name	p-value	Ratio	(%)
EIF2 Signaling	1.57e-28	46/201	22.9
Regulation of eIF4 and p70S6K Signaling	1.08e-08	21/175	12
Axonal Guidance Signaling	9.1e-07	34/483	7
mTOR Signaling	9.57e-07	21/213	9.9
PI3K Signaling in B Lymphocytes	3.92e-06	16/143	11.2

**Fig 7:**
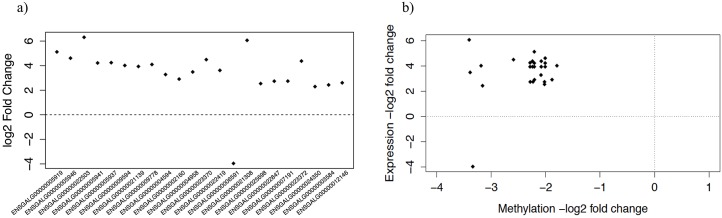
Gene expression of commonly detected genes and expression-methylation relationship. a) Relative gene expression of twenty-two common genes detected by both methods (Annotation of the Differentially Methylated Regions and RNA-Seq analysis) in vaccinated against control birds b) Gene expression and methylation level of its correspondent peaks. The points that perfectly align to the ordinates, are different genes assigned to a singular DMR. Observations with the same gene expression value but diverse methylation levels correspond to several DMRs contained within a gene.

To explore the relationship between DNA methylation and gene transcription, comparison between differentially expressed genes—RNA-Seq analysis—from a previous study [23] and DMR annotated genes, was carried out. We detected 22 common entities from an original list of 173 genes with divergent gene expression. As shown in the Fig 7a, the relationship of expression levels for these genes in vaccinated and control birds was found. Notably, 21 of the 22 common genes were upregulated in the vaccinated group. However, to further explore the relationship between methylation level in promoters and gene expression, we identified the peaks that overlap with promoters (± 5 kb from the TSS), and plotted gene expression against methylation level. From the Fig 7b, we found 17 of those 18 genes had high methylation with low gene expression in the control group, meaning that these genes were up regulated in the vaccinated birds. *ENSGALG00000006591* was the only gene that decreased its expression concurrently with methylation in the vaccinated birds (Fig 7b). Some genes presented more than one annotated DMR, i.e., gene *ENSGALG00000021139* contained the maximum number of DMRs, allocating four in its body and the fifth overlapping the end of the gene. For *ENSGALG00000002160*, *ENSGALG00000005941*, *ENSGALG00000009594*, *ENSGALG00000022847* and *ENSGALG00000023372* two DMRs were identified. The rest of the genes have unique DMRs. In contrast, some of the regions with distinct methylation levels affect not only a single gene promoter region, e.g., *ENSGALG00000005919*, *ENSGALG00000005941* and *ENSGALG00000021139* share the same DMR. For this regard, we only identified 19 unique DMRs, corresponding to the 18 previous reported differentially expressed genes.

## Discussion

Today, DNA methylation is one of the best-recognized epigenetic marks, which are mostly associated with heterochromatin and repressive state of gene [24]. Thus, the identifications of epigenetics marks are of important values on developmental, growth and disease diagnose. So far, there have been several methods applied to DNA methylation analysis [25]. Among them, MBD-Seq constitutes a good alternative for detection of DNA methylation in genome wide studies [21]. The method is cost-effective and not affected by the specificity of antibodies, because it relies in affinity of the protein MBD2. Particularly, MBD-Seq employs different salt concentrations to elute the fragments regarding their methylation density. However, the optimum number of salt cuts depends on the experiment requirement. In the present, three concentrations were enough to cover most of the methylated regions. MBD-Seq requires similar sequencing depth as MeDIP to detect around 70–80% of the CpGs in the human genome [26]. Currently, bisulfite conversion methods (i.e.: MethylC-Seq) are the gold standard for methylation detection but the cost and demanded labor still prohibits its massive use for genome wide screenings [27, 28]. In the ILTV infection, the 1,155 detected DMRs characterize the difference in DNA methylation patterns between control and vaccinated birds. We know that many physiological changes occur after an organism is exposed to an antigen; however, the role of DNA methylation in the complex mechanisms of immunological response is not well understood. Owing to three levels of salts used in the MBD-Seq analysis, the different salt concentration varies binding affinity related to CpG islands density. As expected, high concentration elutes have the less number of detected peaks. This finding agrees with the fact that only few genomic regions presents high CpGs frequency [29]. The numbers of peaks within each concentration were considerable homogeneous, demonstrating the consistency of the applied peak calling method.

As we know, DNA methylation has an main role in cancer and other pathologies [30]. It is also recognized that methylation of promoters leads to inactivation of tumor repressor genes in different cancer types [31]. However, global hypomethylation promotes genomic instability, producing aberrant cell transformations observed in cancer [32, 33]. In this research, we found that DNA methylation globally decreased in vaccinated birds. The data coincided with early reports in Marek’s Disease and suggests that for individuals to be capable to respond to an antigen inoculation, genes involved in the immunological response should be activated [34, 35]. Nevertheless, in order for this to happen repressive marks as DNA methylation must be removed. Although this occurred in most of the DMRs, 451 regions presented an increment in DNA methylation. These regions can be related to the genes that actively repress the immune machinery under normal conditions, but are silenced to release the immune response in other circumstances. Notably, identified DMRs were in average 1,215 bp long. However almost 60% were shorter than 1,000 bp and only 1% could reach 4,000 bp or more (Fig 3a). This suggests that methylation in relatively small fragments of DNA is sufficient to regulate gene activity and ultimately influence biological functions. Visualization of the DMRs’ chromosomic distribution shows that the largest number of DMRs was assigned to unspecific locations in the random chromosome. Chromosome 1 allocated the second larger number with 172 divergent methylation fragments, and chromosomes 16 and 22 with no more than 5 DMRs (Fig 3b). Although the DMR numbers are not normalized regarding chromosome length, we can see the bigger chromosomes contain the more numbers of DMRs.

Promoter are normally localized close to their respective gene, extending from 100 to 1000 bp [36]. Also, evidence showed that gene promoters display chromosome-specificity and reveal chromosome territories [37]. Accordingly, we analyzed the distance of the DMR to the TSS of the closest gene, considering the transcription direction. We found most of the DMRs surrounded the TSS within 10 kb. The second largest group allocating almost 250 DMRs are upstream the TSS, confirming previous findings [38, 39]. Interestingly, bidirectional promoters can trigger transcription in both directions. Moreover, non-coding transcription at promoters can influence protein-coding genes, suggesting a new level mechanisms of regulation [22]. In the present we detected 165 genes that are located closely to bidirectional promoters. Further studies should be performed to analyze the relationship between them and their products, to determine whether they display co-regulation or not, and to reveal more details about transcription regulation in this specific case. Almost 50% of the DMRs reside inside genes, the second largest group is “upstream” followed by the “downstream”. The chicken genome is well annotated comparing to other agricultural species, thus small genes as microRNA are included in the annotation [40]. Some of these small RNAs are totally contained in the DMRs. The components of this group could constitute another layer of gene regulation, the post-transcriptional regulation mediated by microRNAs.

Although vertebrate genomes normally show depletion in CG sites, many regions allocate CpG islands [41]. Portions of the genome, that range from 300 to 3000 bp with a high content of G+C and high frequency—more than expected by chance—of CpG dinucleotides are defined as CpG islands. Usually they contain at least 200 bp and a GC content more than 50% and an observed-to-expected CpG ratio exceeding 60% [42]. CpG islands were associated with the 5’ end of all housekeeping genes and many tissue-specific genes, and some 3’ end of tissue-specific genes [41]. Small number of genes has both 5’ and 3’ CpG islands, distant by several kilo bases of DNA depleted in CpG dinucleotides [43]. Methylated cytosine tend to transform into thymine thus CpG sites are rare in vertebrates; this explains why CpG islands turn to be most of the time unmethylated. In case methylation occurs, it happens in CpG island shores rather than the islands themselves [21]. Methylation of CpGs in promoters’ CpG islands has been associated with a repressive gene expression.

Interestingly, in functional analysis cancer related pathways appeared in the first four top networks, although ILT lacks tumors compared to Marek’s disease, suggesting that genes affected by DNA methylation suffer aberrant cell regulation with oncogenic outcomes. Other networks based on the score are relevant to RNA post-transcriptional modification, humoral immune response, cellular assembly and organization, immunological and inflammatory diseases, protein synthesis, molecular transport and cell cycle. Most of them also associate with the immunological response after vaccination. Notably, among the canonical pathway analysis, the most significant was eukaryotic translation initiation factor 2 (EIF2) signaling. Protein synthesis requires various translation factors to initiate the process [44]. EIF2 is a GTP-binding protein that accompanies the initiation of met-tRNA onto the ribosome and participates in the recognition of the translational start site. Different stimuli can influence EIF2, which finally modulate mRNA translation. Phosphorylation of EIF2-a terminates global translation and causes apoptosis. The next significant pathways are regulation of EIF4 and p70S6 Signaling. Since the recruitment of mRNA to the ribosomes is modulated by EIF4, several stimuli, such as cytokines and growth factors PI3K, PDK1, AKT and mTOR, control EIF4 and p70S6K by phosphorylation cascades [45]. Due to mTOR producing phosphorylation in many different targets they are related to initiation of protein synthesis and reduction of protein translation [46]. Most importantly, mTOR centrally modulates proliferative signal transduction, it was nominated as the ideal target for cancer treatment [47].

As we know, cell migration is critical and occurs during immunological response after vaccination, especially for ILT. In terms of functions of Ephrin B signaling and Phosphoinositide-3-Kinases (PI3K) both modulate several processes such as cell growth, survival, differentiation, metabolism and migration. Therefore, disruption of the PI3K signaling in the immune system could cause immunodeficiency whereas unrestricting signals produces leukemia or autoimmune diseases [48]. Considering all pathways discussed above, the results suggests that a more exhaustive analysis of the components is necessary for control of ILT infection.

In a combinational analysis between transcriptome from RNA-Seq and the DMR regions, we identified some immune-related genes, such *CD74* (*ENSGALG00000004594)*, *B2M* (ENSGALG00000002160) and *LV1L2*, (ENSGALG00000021139). Among them, *CD74* encoded a MHC II protein acting as a chaperone modulating antigen presentation and serving as a receptor in the cell surface for the cytokine macrophage migration inhibitory factor (MIF) [49]. Interestingly, B2M (Beta 2 Microglobulin), codes for a protein founded in the serum associated with the MHC I heavy chain [50]. B2M mutation causes hypercatabolic hypoproteinemia while the protein coded by *GGA*.*47846* interacts selectively with protein complex to counteract the effect of the antigen [51]. Similar to *GGA*.*47846*, gene Immunoglobulin lambda chain V-1 region-like 2 *(LV1L2*) also participates in the immune response. Gene *5_8S_rRNA* encodes a noncoding RNA found in the ribosome that participates in protein synthesis [52]. Definitely, a lot of questions remain unexplainable regarding the role of DNA methylation during the immune response after vaccination. However, the goal of this study was to describe the different DNA methylation profiles between these two conditions.

In summary, we reported the first methylome profile related to vaccination in ILTV infection in birds. We found the global DNA methylation was decreased in vaccinated birds. In the identified 1,155 DMRs, we found 451 hypermethylated and 704 hypomethylated DMRs in vaccination experiment, respectively. After annotation, the identified biological pathways were mostly involved in the immune response post vaccination. Meanwhile, we also explored the relationship between DNA methylation and gene expression. The present study constitutes a first detailed DNA methylome profile in immunized chicken with ILT (CEO) vaccine, which open opportunities for novel targets in prophylaxis and further provide new insights for future studies regarding the role of epigenomic marks during immunological response.

## Material and Methods

All chickens were obtained from Charles River Laboratories. They consist in ten, 15 day-old White Leghorn chickens that were randomly assigned to groups of five birds each. After 6 days, the control group received only sterile vaccine diluent (50 μl per nostril and 50 μl per eye, for a total dose of 200 μl per animal) and then allocated in a separated animal room. Later, the vaccine was prepared following the manufacturer’s recommendations and the vaccination was performed in the second group. The procedure was the same as for the control group, excepting for the viral load (3.3 x 10^3^ pfu). The vaccinated birds were placed in an animal isolator in another suite.

### Ethics Statement

All animal experiments were conducted following NIH guidelines for housing and care of laboratory animals and in accordance with The University of Maryland at College Park (UMCP) regulations after review and approval by the UMCP Institutional Animal Care and Use Committee (permit number R-08-62).

### Vaccine Description

We used the modified live chicken-embryo origin vaccine, Trachivax and prepared the vaccine solution according to the manufacturer’s instructions for ocular administration [53]. The vaccine was titrated in kidney cells obtained from 20-day-old pathogen free chicken embryos. Dilutions of the vaccine ranged from 10^–1^ to 10^–5^. Four replications for each dilution were inoculated into 12-well plates with 100% chicken-embryo kidney cell confluence for 1 hour. Immediately, cells were washed with sterile phosphate buffered solution (PBS) and covered with 0.8% methylcellulose in MEM D-Valine media (Promo Cell, C-75100), containing 2% fetal bovine serum (HyClone Laboratories, SH30071), 1% sodium pyruvate (Invitrogen, 11360070) and 1% L-glutamine (Sigma-Aldrich, G7029). After 96 hours of incubation at 37°C plaque forming units were counted for viral quantification.

### Symptoms and Clinical Scoring

Birds were monitored daily, starting the day of inoculation. Any observed abnormality was accurately recorded and the chickens were classified according to the following criteria: 0 (normal), 1 (mild eye inflammation), 2 (oculo-nasal discharge), 3 (coughing and sneezing) and 4 (expectoration of bloody mucous or respiratory distress)

### Sample Collection

At 6-day post infection, all chickens were humanely euthanized following the IACUC guide. Immediately, thoracic cavities were accessed and the trachea carefully dissected. Employing sterile instruments for individual samples, each trachea was completely incised longitudinally and the mucosa exposed. Finally, the tracheal mucosa was scraped from the larynx to the syrinx using a sterile disposable scalpel. Samples were placed in individual identified tubes filled with RNA later solution (QIAGEN, 76106) at -80°C for DNA or RNA extraction.

### DNA extraction and MBD-Seq library preparation

Genomic DNA from five samples of each group was extracted using the Wizard Genomic DNA purification kit (Promega, A1120). DNA concentration was measured by the Qubit dsDNA Broad-Range Assay (Invitrogen, Q32850). Two DNA samples were randomly selected from each group and named as Control 1, Control 2, Vaccinated 1 and Vaccinated 2, respectively.

MBD-seq method was used to identify methylated DNA regions. MethylCap kit (Diagenode, C02020010) was employed to obtain DNA containing methylated CpGs. Firstly, DNA was extracted and dissolved to reach 0.1μg/μl for a final volume of 40 μl in a 1.5 ml tube. Then DNA was sheared into 300–500 bp fragments using the Bioruptor Sonicator and checked on agarose gel to visualize the size of the resultant segments. Secondly, 141.8 μl of capture reaction mix without MethylCap protein, but containing 12 μl of sheared DNA was prepared. From this preparation, 119 μl of capture reaction mix was incubated with 1 μl of diluted MethylCap protein at 40 rpm on a rotating wheel for 2 hours and at 4°C to let the interaction occurs. The rest (22.8 μl) was used later as input sample. Magnetic beads (coated with GSH) captured methylated DNA. After, unbound DNA was washed off and DNA elutes collected. For the elution, 150 μl of low, medium, and high concentration buffer was used serially per each capture. All fractions and input were purified using the MiniElute PCR Purification Kit (QIAGEN, 28006). Finally, qPCR (iCycler iQ PCR system, Bio-Rad) was performed in duplicates for each sample to test the enrichment efficiency. Tested primers are listed in S1 Table. Method 2^-ΔΔCt^ was applied to determine relative fold enrichments; comparing enrichment values of a positive *TGFB3* to a negative primer pair *MON2*, between experimental (methyl DNA) and reference (input DNA) samples.

The library for sequencing was constructed as follows. NEBNext End Repair Module (NEB, E6050S) was used for the end repair of the fragmented methylated DNA. Then a 3’ A was added using DNA Polymerase I, Large (Klenow) Fragment (NEB, M0210L). Also, a pair of Solexa adaptors (Illumina) was ligated to the repaired ends by T4 ligase (Promega, M1801). Filtration in a 2% agarose gel was used to select fragments (DNA plus adaptors) from 200 to 500bp. PCR enriched purified DNA templates by using Phusion Hot Start High-Fidelity DNA Polymerase (NEB, M0530S). After purification, DNA quality was examined. The DNA library was diluted and the concentration double-checked using the Qubit assay (Life Technology, Q32850). Finally, we performed sequencing analyses in the Solexa 1G Genome Analyzer (Illumina) following manufacturer protocols.

### MBD-Seq Data Analysis

Sequence files were examined for quality assurance. After an adequate quality confirmation, files were aligned to the galGal3 (WUGSC 2.1) reference genome obtained from the UCSC browser (http://genome.ucsc.edu). For the alignment process, Bowtie (Ultrafast, memory-efficient short read aligner) was employed [54]. Original fragments consisted in 50 nucleotides although the first 15 5’ nucleotides of each fragment were trimmed to maintain high sequence quality. For data manipulation, filtration and format conversion a combination of procedures available in SAMtools and BEDtools were applied [55, 56]. An important step that should be considered is the removal of duplicated reads, which has been achieved using the bRemoveDuplicates option included in the DiffBind package. This action will affect posterior procedures and is critical for the analysis method that we used.

The peak-calling step was applied individually for each sample using Model Based Analysis of ChIP-Seq (MACS) [57]. The software empirically models the shift size of the tags and employs a dynamic Poisson distribution to account for local bias, making the predictions more reliable. Identification of the Differentially Methylated Regions (DMRs) was accomplished implementing the DiffBind R package [58]. It computes differentially bound sites using affinity data. The set of peaks identified by MACS and the bam files containing aligned reads for each sample, was the input for DiffBind. The program creates a matrix with the consensus peaks; for this case it was obtained with a “minimum overlap” of 2, determined by the number of replications in the experiment. After creating a contrast between conditions and considering concentration as a block effect, DiffBind runs an edgeR analysis, which is an empirical Bayes method [59]. For normalization, the default method TMM (Trimmed Mean of M-values) that subtracts the controls reads and considers the effective library size (reads in peaks), was applied. The threshold utilized was 0.1, for False Discovery Rate (FDR).

For the genomic annotation of the DMRs, the software ChIPpeakAnno was used [60]. ChIPpeakAnno provides information about the overlaps, relative position and distances for the inquired feature. The annotation information was obtained from biomart, using Ensembl 70 in the archive site. Dataset “ggallus_gene_ensembl” corresponds to WASHUC2 (galGal3), the genome used for alignment. The CpG island annotation was retrieved from UCSC web browser. For the enrichment analysis, genes that were annotated based on the nearest TSS or overlaps with the peaks were considered. An Interactive Pathway Analysis (IPA) was performed using the Ingenuity software (http://www.ingenuity.com). The analysis generated an extensive report, but the most valuable are: networks, diseases and disorders, molecular and cellular functions, physiological system development and function, and canonical pathways.

### Bisulfite Sequencing for MBD-seq Validation

Sodium bisulfite conversion reagents were used to treat 500 ng of DNA (EZ DNA Methylation Golden Kit, ZYMO Research, D5005) following the standard protocol provided by the manufacturer. PCR primers were designed using MethPrimer (http://www.urogene.org/cgi-bin/methprimer/methprimer.cgi) and listed in S1 Table. Firstly, equal amounts of DNA from five samples of each group (vaccinated and control) were pooled together, serving as a template for the bisulfite conversion and the bisulfite PCR. Then, PCR resultants were purified using QIAquick Gel Extraction Kit (QIAGEN, 28704). The purified PCR products were ligated to pGEM-T Vector (pGEM-T Vector System I, Promega, A1360), transformed to DH5α competent cells (Z-Competent E. Coli Cells—Strain Zymo 5α, ZYMO Research, T3007), and screened for successful insertions (blue-white selection) after incubation at 37°C overnight. In the next step, ten white colonies from each group were cultured overnight in a 37°C shaker. Plasmid DNA was isolated using Zyppy Plasmid Miniprep Kit (ZYMO Research, D4036). M13 reverse primer and BigDye Terminator v3.1 Cycle Sequencing Kit (Applied Biosystems, 4337456) was employed for sequencing in the ABI 3730 machine. Bisulfite sequencing results were analyzed by QUMA (http://quma.cdb.riken.jp) and DNA methylation level for each region and group, obtained.

## Supporting Information

S1 Table PCR Primer sequences used for MBDSeq validation. (DOCX)

S2 Table Descriptors and statistics of differentially methylated regions (DMRs) identified by MBDSeq method. (XLS)

S3 Table Annotation of the differentially methylated regions (DMRs) regarding Ensembl genes. (XLS)

S4 Table Identified bi-directional promoters and its corresponding Ensembl annotation. (XLS)
