# Supplementary material for: Methylome Analysis in Chickens Immunized with Infectious Laryngotracheitis Vaccine
Source: PLoS One. 2015 Jun 24;10(6):e0100476. doi: 10.1371/journal.pone.0100476 (PMC4481310; doi:10.1371/journal.pone.0100476)
Supplement: S1 Table — (DOCX) [file pone.0100476.s001.docx]

**S1 Table. PCR Primer sequences used for MBDSeq validation**

| (q)PCR experiment | Primer name | Sequence | Product Size (bp) | Annealing Temperature (℃) | Related RefSeq Genes |  |
| --- | --- | --- | --- | --- | --- | --- |
| MBD_Check | TGFβ3 | F: 5' 3'  R: 5' 3' |  | 60° | *TGFβ3* |  |
| MBD_Check | MON2 | F: 5' 3'  R: 5' 3' |  | 60° | *MON2* |  |
| MBD_Validation | MBD1 | F: 5'TGAGGAGTTGAGTTTTTTATGTTTG3'  R: 5'AACAAACACACCCCAACAATACTA3' | 510 | 60° | No^*^ |  |
| MBD_Validation | MBD3 | F: 5'TAAATTGTGAGAGGTTGGTTTAGTA3'  R: 5'ATAAAAAAATCAATTACCTCCCTCC3' | 518 | 60° | No |  |
| MBD_Validation | MBD4 | F: 5'AAAAGTTATATTTTGTTGGGTTGTTG3'  R: 5'CAACATTTTATTACCTTTCCATCAAA3' | 500 | 60° | *ENO1* |  |
| MBD_Validation | MBD5 | F: 5'TTTTGTTTTTAGATGTTGTGAGTTGA3'  R: 5'TATTATTACAATACAAACCACCCCC3' | 504 | 60° | *RFC4* |  |
| MBD_Validation | MBD7 | F: 5'GAGGTTGGATGTGGTTTTGG3'  R: 5'CACGACTAAATACGCGACCA3' | 631 | 60° | *RPS12* |  |
| MBD_Validation | MBD8 | F: 5'TTTTGGAATCGAGTTTTGTGA3'  R: 5'CAAAACCTCAACCACCCTAAT3' | 716 | 60° | *KPNA2* |  |
| MBD_Validation | MBD13 | F: 5'GTTGTTATGAAGTAATTTGAAGGAGGTGTT3'  R: 5'TCCATTCAATAAAACAAACCTATAATCTCA3' | 371 | 60° | *PABPC1* |  |
| MBD_Validation | MBD14 | F: 5'ATTTTTGTTTTTGGAGTAGTGTATTTG3'  R: 5'AACTTTTAACTCTATAAAATCTCCTTTTCC3' | 444 | 60° | *RPL6* |  |
| MBD_Validation | MBD15 | F: 5'TTTGTGTTTGTGGTTGTTTGGGGTT3'  R: 5'ACAACACTTTTAATTCCCACCCCACCTA3' | 318 | 60° | No |  |
| MBD_Validation | MBD16 | F: 5'TGTGTATTTGGGGTATTATTTATTGATTTT3'  R: 5'TCAACCACTTATAAAAACTCTCATTTCACT3' | 474 | 60° | No |  |
| MBD_Validation | MBD18 | F: 5'TGTAGAAATGTTTTTTTTGGGGAGA3'  R: 5'AACAAAACCACTAACCAATACAATCTTTAA3' | 348 | 60° | *STMN1* |  |
| MBD_Validation | MBD19 | F: 5’GGAGGTTTTATAGGGGAATTTTTTTT3’  R: 5’TTACCCACAAATTAAACTCTAACCTTCTTA3’ | 370 | 60° | *FTH1* |  |
| MBD_Validation | MBD22 | F: 5’GAGGGGGAGTTTTATTTGTTGTTAG3’  R: 5’CACAAAAAAAATTTCTATACAAATACCC3’ | 340 | 60° | No |  |
| MBD_Validation | MBD23 | F: 5’TTAGGAATTTTTGTTTGTGGGATTTTAGTT3’  R: 5’ATCTCTACAACCCCTACATCACCTC3’ | 619 | 60° | *NCOA1* |  |
| MBD_Validation | MBD24 | F: 5’GAGGTGATTGTAGATGTTGGATTTGATAAG3’  R: 5’AACATCAACTCCCCAAAAAACACAC3’ | 363 | 60° | No |  |
| MBD_Validation | MBD26 | F: 5’AAGGGATGGAGTGGTTTAGTTTTTTA3’  R: 5’ACTATTCCCCTCCCCTAACTATCATTATAC3’ | 375 | 60° | No |  |
| MBD_Validation | MBD27 | F: 5’GGGGAGAATGAGTTTTTTGAGGATTATTTT3’  R: 5’ATAACTAACCATCTCACCTCCTCCATCACT3’ | 506 | 60° | No |  |
| MBD_Validation | MBD28 | F: 5’TAGTTAGGTTTTGAGGGGAAGGAGGAG3’  R: 5’CCCAAATAAAACAACTAACAATCTCCTAAA3’ | 536 | 60° | No |  |
| MBD_Validation | MBD33 | F: 5’AGAGAGGGAGGTGAGTTTTTTTGGAGTATA3’  R: 5’TTTCAACTCTCCCTACCACCAAATCC3’ | 493 | 60° | *TK1* |  |
| MBD_Validation | MBD34 | F: 5’TTGTTGTTTTGTGGAGATGGGGTTT3’  R: 5’TCCCAACACTACTACATCCCCAACC3’ | 428 | 60° | No |  |
| MBD_Validation | MBD35 | F: 5’GGGGTTGGAGTAGGGGAGGGTTTAT3’  R: 5’CCAAAACAAAAAACACCAAAACAAC3’ | 680 | 60° | No |  |
| MBD_Validation | MBD36 | F: 5’GGTAGTTGTTTTTTTAAGGTGGGT3’  R: 5’ATCCTCTCTTTACTCAATCTAACCCC3’ | 372 | 60° | No |  |

^*^No means: no chicken refSeq genes overlap with the PCR sequence of this MBD region.
